# Human genome-microbiome interaction: metagenomics frontiers for the aetiopathology of autoimmune diseases

**DOI:** 10.1099/mgen.0.000112

**Published:** 2017-04-26

**Authors:** Aycan Gundogdu, Ufuk Nalbantoglu

**Affiliations:** ^1^​ Erciyes University School of Medicine, Turkey; ^2^​ Genome and Stem Cell Center (GenKok), Erciyes University, Turkey; ^3^​ Department of Computer Engineering, Erciyes University, Turkey

**Keywords:** genome-microbiome interaction, metagenomics, autoimmune disease

## Abstract

A short while ago, the human genome and microbiome were analysed simultaneously for the first time as a multi-omic approach. The analyses of heterogeneous population cohorts showed that microbiome components were associated with human genome variations. In-depth analysis of these results reveals that the majority of those relationships are between immune pathways and autoimmune disease-associated microbiome components. Thus, it can be hypothesized that autoimmunity may be associated with homeostatic disequilibrium of the human-microbiome interactome. Further analysis of human genome–human microbiome relationships in disease contexts with tailored systems biology approaches may yield insights into disease pathogenesis and prognosis.

## Abbreviations

GWAS, genome-wide association studies; MGWAS, metagenome-wide association studies.

## Impact Statement

Recent studies on human genome–microbiome interactions (metagenome-wide association studies, MGWAS) showed that the presence or abundance of certain microbiome components is associated with structural variations in the human genome. Here, with a focused look, we relate these recent findings to a relevant human disease class. A key observation in these studies is that differences in the microbiome composition are mostly associated with variations in human genome that have previously been associated with autoimmune diseases. This article aims to draw attention to the necessity for further studies that have been carefully designed to reveal the diseases that are related to microbiome dysbiosis, as well as the factors contributing to dysbiosis. Metagenome-wide association studies may enable the development of microbiome-based diagnostics and therapies.

## Introduction

Two ground-breaking projects in the early genomic era changed the landscape of health sciences: the Human Genome Project (1990–2003) [1–3] and the Human Microbiome Project (2008–2012) [4, 5]. The early promise of the Human Genome Project was to be able to annotate the genetic variations associated with human diseases, based on comparisons with the published reference genomes. The scientific community has gradually diverged from that view given the findings of genome-wide association studies (GWAS), in which genetic variations have been investigated in large cohorts [6]. Genetic variations, even when considered in epistatic, multi-gene models, are unable to account for complex diseases. Indeed, an extensive GWAS, which employed relatively large cohorts, could explain no more than 3 % of metabolic disease cases using predictive models of 30 gene loci that had been inferred to be associated with the corresponding disease [7]. The new perspective on chronic diseases, with the exception of Mendelian diseases with relatively large effect sizes, is that many are driven by complex and systems-level disorders. Accordingly, multiple genes, as well as non-genetic or non-epigenetic environmental factors, play crucial roles [8]. As certain complex diseases emerge as chronic impairments, where impairments of the immune system and the microbiome converge to create homeostatic imbalance, hologenomic interactions must be investigated.

## Microbiome as quantitative trait

Pioneering studies that jointly considered the human genome and microbiome reported that the presence or abundance of certain microbiome components is associated with the structural variations in the human genome [9, 10]. For instance, Blekhman *et al*. gleaned human genome contamination in the Human Microbiome Project data and detected specific host genetic variations in the context of each individual microbiome using bioinformatics approaches [9]. Their report was the first GWAS on human genome–microbiome associations. Strikingly, they found that those variations associated with changes in the microbiome were mostly observed in the loci previously associated with autoimmune diseases. It has been postulated that autoimmune diseases develop after exposure to environmental triggers in patients with a genetic predisposition. Immune system pathway genes, such as HLA,CRF4, PTPN22, TBX21, STAT4, IRF4,IRF5, CD247, BANK1, BLK, IRAK1, FOXP3 and TNFAIP3 are commonly associated with several autoimmune diseases (e.g. rheumatoid arthritis, Coeliac disease, type I diabetes, psoriasis, Crohn’s disease and systemic sclerosis) [11, 12]. Many autoimmune diseases, which involve systemic disorders, could be classified as pleiotropic disorders. Moreover, recent host genome–microbiome association studies revealed that these genes were among those associated with microbiome composition. According to a study conducted by Volkmann *et al*., which considered the gut microbiota of 17 systemic sclerosis patients and an equal number of healthy controls in order to reveal potential pathobionts, the differentiation of the gut microbiota associated with the disease shows significant similarities with the gut dysbiosis of Crohn’s disease [13]. This observation implies that autoimmune diseases may share characteristic host-microbiome variations. Furthermore, the pleiotropic nature of autoimmune diseases (i.e. a single variation may be associated with multiple phenotypes) supports the view that the autoimmunity reactome has certain properties which should be investigated at the systems level, in a multi-omic investigation of human genome–microbiome metagenomes.

## The homeostasis of a superorganism

The human microbiome corresponds to a complicated ecosystem; it is a large population that forms a symbiotic super-organism in combination with the human organism. As there are 10-fold more microbial cells than *Homo sapiens* cells in the human body, the human microbiome, which contains millions of genes, has a far greater proteome-coding and metabolome-producing potential than the human genome, which harbours only around 20 000 genes [14]. Environmental factors shape the microbiome composition, including the mode of delivery at birth and the inherited maternal microbiome [15]; diet [16, 17]; living space [18]; social interactions [19]; and exposure to xenobiotics, pathogens and parasitic organisms [20, 21]. In addition, the interaction of the gut microbiome with its host directly shapes the composition [22]. The currently illuminated part of this interaction system mainly corresponds to the immunity metabolisms. Recently revealed interactions between the microbiome and the immune system show that they exist in a delicate homeostasis. Tipping of the equilibrium by perturbations stemming from either, or both, of the symbiotic partners might result in disturbances of processes related to inflammation, autoimmunity, metabolism, neurodegeneration and the development and progression of cancer [23–25]. Gut microbiome dysbiosis might lead to a decrease in the activity of critical members or metabolic processes, resulting in autoimmune disease (with the microbiome being the primary cause of pathogenesis). Conversely, the primary driver of the aetiopathology of immune disorders may be the inability to distinguish between commensal and pathogenic components of the microbiome, induced by environmental triggers in genetically predisposed individuals (host genetics are the primary cause of pathogenesis). Although the pathogenesis of a large proportion of autoimmune diseases is currently unclear, the involvement of the human microbiome in both scenarios above raises an important question: can the gut microbiome be used as a diagnostic biomarker or a therapeutic target for the prevention and treatment of autoimmune diseases? The human genome is static and, except for specific modifications (e.g. genome editing), cannot be manipulated over the lifetime of an individual. The human microbiome, on the other hand, is the plastic ‘other genome’ of the human super-organism that could be shaped or even reassembled by probiotics, antibiotics, diet, vaccination and transplantation techniques. This potential makes prospective microbiome-targeted therapies attractive. The promise of this approach has been observed when the manipulation of commensal bacteria caused remission of rheumatoid arthritis in mice [26]. Similarly, a healthy microbiome prevented multiple sclerosis in mice, while germ-free mice in the same conditions developed the disease [27]. Further studies should be conducted to reveal the nature of the disease-related dysbiosis and the factors affecting it, in order to enable the development of diagnostics and therapies based on the microbiome.

## A complex interactome requires sophisticated analysis

**Fig. 1.**
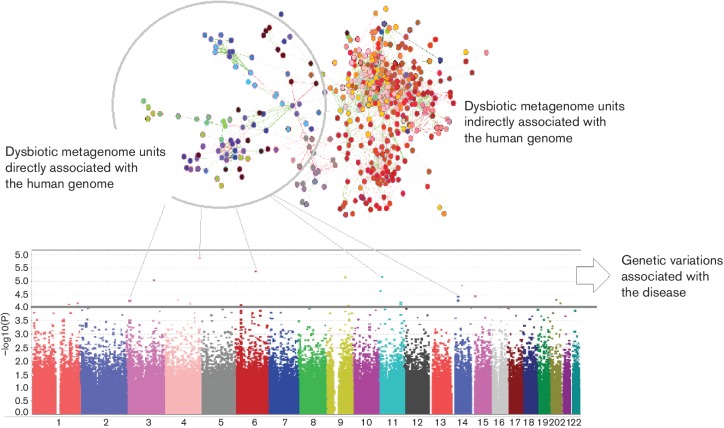
A systems biology approach to MGWAS. Fusion gene-disease interaction networks are compiled from the GWAS Catalog and the metagenomic unit (e.g. gene, ontology group, metabolic pathway or operational taxonomic unit) co-occurrence network built using metagenome data. Feature selection by machine learning wrappers suggests associations based on variations in the metagenomic units to infer direct and indirect associations between genomic variations and the microbiome in the context of the disease.

While the findings of the microbiome-GWAS studies shed light on the association between human immunogenetics and the human microbiome, they are early discovery attempts in the genome–microbiome multi-omic field, which requires further development. The current paradigm is built on a naive approach, which models the microbiome as a quantitative trait. This is mainly through the quantification of the microbial community via α or β diversity metrics or evaluation of the relative abundance of an operational taxonomic unit. However, in the context of the novel idea of the super-organism, or the second genome, the microbiome should be treated as an extension of the human genome rather than a quantitative trait. Previous 16S rRNA gene sequencing studies were designed to taxonomically profile the microbiome. In order to explore its functional diversity and construct metabolic models within a microbiome, a shotgun metagenomics approach is required. Sequencing the genetic content in this manner would allow functionally classified genes to be grouped and gene networks to be constructed in order to form human genome–microbiome interaction networks. Early versions of the related metagenomic unit co-occurence networks, such as the metaHIT catalogue [28], are already available. A systems biology model, which integrates metagenomic gene networks with the disease associations map in the GWAS Catalog [29], has the potential to suggest direct and indirect genetic variation–microbiome associations in the context of diseases. Fig. 1 depicts a model constructed from this perspective. Moreover, hologenome theory suggests that a host co-evolves with its microbiome. From this point of view, strain-level divergences that alter the function of certain microbial species could be expected. The study of strain-level differentiation, which is also interesting in the context of diseases, can only be resolved by employing shotgun metagenomics. Recent advances in metagenomics informatics enable careful taxonomic profiling at the strain level [30–32]. Parallel to the current paradigm for microbiome analytics, GWAS that associate genomic variations with quantitative traits depend on conventional statistical approaches and single gene/single-nucleotide polymorphism -quantitative trait correlation estimations. However, the majority of diseases and other attribute phenotypes are epistatic, meaning that the overall phenotype is the net effect of multiple genes with small effect sizes. Unfortunately, the conventional GWAS approach has weak sensitivity and is able to detect only genetic variations with large effect sizes [33].

The introduction of machine learning and data mining applications, in which multiple gene variations are associated with phenotypes, would allow epistatic inferences to be made with greater statistical accuracy, according to retrospective studies [6]. By assuming that either individual microbiome elements or the community diversity are quantitative traits, the current GWAS methodology to determine genome–microbiome associations ignores the potentially epistatic nature of the symbiosis. Both of the recent studies conducted by Blekhman *et al*. [9] and Davenport *et al*. [10] followed the conventional one gene-one quantitative trait GWAS approach. However, human genome–microbiome relationships are thought to be both epistatic and pleiotropic [34]. On the other hand, since the sampling and sequencing of metagenomes require substantial effort and costly procedures, current cohorts are orders of magnitude smaller than the operational ranges of GWAS. Thus, the observed associations likely appeared due to their large effect sizes. Importantly, many genetic associations may be ignored by underpowered and insensitive analyses. In order to mitigate this problem, enrichment via combining associated variances on the same pathways is suggested. This approach was observed to provide further associations [9]. Nevertheless, the improved approach still follows the GWAS paradigm, in which very low *P*-values are needed before associations can be proposed. As an alternative, predictive models supported by machine learning concepts can be proposed to statistically quantify genome–microbiome associations. Feature selection with learning machines has enabled detection of cross-validated disease biomarkers at the current metagenome-sampling cohort size [35]. Once features comprised of groups of single nucleotide polymorphisms and bags of metagenomic genes are selected, predictive models (e.g. Support Vector Machines [36], Random Forest [37]) can be constructed to perform cross-validation tests that quantify the significance of the associations. For the aforementioned reasons, it is indispensable to build future projections on metagenome-wide association studies (MGWAS) bioinformatics, assessing multiple gene–multiple phenotype associations in a machine learning context. It is important to consider that, while very sensitive and powerful enough to infer associations from a relatively small number of instances (e.g. the current cohort size of metagenome projects), machine learning approaches are vulnerable to overfitting and suggesting false positives, especially in the context of a large number of variables, as in the case of MGWAS [38]. Therefore, careful bioinformatics strategies should be developed in order to make sensitive, yet sufficiently specific, MGWAS analyses to reveal further human genome–microbiome interactions. It should be noted that the top-down approach of systems biology via omic technologies only captures snapshots of a complex living system. While this *in silico* support is a valuable hypothesis proposal paradigm, the results (e.g. detected biomarkers or suggested biological models) should always be subjected to *in vivo* validations. For example, the precise microbiome targets suggested by the multi-omic studies should be tested in knockout animal experiments, as the early phase of metagenomic drug discovery studies. Moreover, independent autoimmune disease cohorts should be enrolled for the validation of the discovered, non-invasive diagnostic biomarkers.

In the future, it will be possible to conduct multi-omic studies with larger cohorts as sequencing technologies provide lower cost and higher throughput solutions. This will generate more insightful data, with enhanced generalization ability and statistical power. Given that, focussing on longitudinal study designs may provide further insights, not only because they would capture temporal changes along with disease prognosis, but also because metagenomic units associated with human genetics might become more detectable. Recent twin studies have reported that the taxa that are controlled by host genetics tend to be temporally more stable taxa that were not associated with host genetics [39]. Consideration of the temporally low-variance metagenomic units could add another dimension to the computational prediction of genome variation–microbiome associations.

## Conclusion

Based on the recent literature, it is plausible to infer that the associations between variations in human immune system pathway genes and certain human microbiome taxa might imply the interplay of human genome–microbiome in the pathogenesis of autoimmune diseases. Thus, multi-omic studies, in which the microbiome and the human genome are sequenced jointly, involving autoimmune patients and corresponding healthy cohorts will dramatically improve our understanding of complex diseases. To this end, machine learning-based systems biology may be useful, given that the current metagenomic cohorts, ranging from a few hundred to a few thousand subjects [10], are statistically underpowered to conduct conventional GWAS, which confines the discovery of associations to only highly specific mechanisms.
